# Mind Melds: Verbal Labels Induce Greater Representational Alignment

**DOI:** 10.1162/opmi_a_00153

**Published:** 2024-08-09

**Authors:** Ellise Suffill, Jeroen van Paridon, Gary Lupyan

**Affiliations:** University of Wisconsin-Madison; University of Vienna

**Keywords:** categorization, labels, alignment, language and thought, sorting

## Abstract

What determines whether two people represent something in a similar way? We examined the role of verbal labels in promoting representational alignment. Across two experiments, three groups of participants sorted novel shapes from two visually dissimilar categories. Prior to sorting, participants in two of the groups were pre-exposed to the shapes using a simple visual matching task designed to reinforce the visual category structure. In one of these groups, participants additionally heard one of two nonsense category labels accompanying the shapes. Exposure to these redundant labels led people to represent the shapes in a more categorical way, which led to greater alignment between sorters. We found this effect of label-induced alignment despite the two categories being highly visually distinct and despite participants in both pre-exposure conditions receiving identical visual experience with the shapes. Experiment 2 replicated this basic result using more even more stringent testing conditions. The results hint at the possibly extensive role that labels may play in aligning people’s mental representations.

## INTRODUCTION

Linguistic communication requires conversants to have some degree of conceptual alignment (e.g., Pickering & Garrod, 2021). One person’s meaning of “Pass the salt” or “red car” must be sufficiently shared with other members of their speech community. If it were not, we would encounter regular failures in communication. And although communication failures certainly exist (e.g., Albert & de Ruiter, 2018; Schober et al., 2018) by and large we are able to make ourselves understood and to understand others (Moore & Carling, 1988)—at least when communicating with those from our speech community. But where does this conceptual alignment come from and might language itself play a role in establishing it?

One perspective is that our conceptual representations and word meanings^1^ are aligned because people are biologically similar, have similar learning mechanisms, and live in broadly similar environments. For example, because no human sees ultraviolet light, there is no risk that some people’s meaning of “blue” extends into the ultraviolet range, thereby frustrating communication with people whose meaning of “blue” does not. Likewise, common cognitive constraints ensure that there is little risk of someone’s meaning of “dog” denoting only dogs seen at 3:14 pm from the side (contra Funes, the fictional character who could not categorize, Borges, 1999). Such peculiar specificity would violate basic human categorization (Rosch, 1978; Shepard, 1994). This account presumes that our conceptual representations (at least of things that do not require formal instruction) are *already* sufficiently aligned; this prior alignment may even be what makes linguistic communication possible in the first place (Fodor, 1975). This perspective is a common starting point in theories of language learning that view children as mapping words onto pre-existing and largely shared concepts (Bloom, 2002; Pinker, 1994; Snedeker & Gleitman, 2004; cf. Lupyan, 2016; Wojcik et al., 2022).

Another possibility is that alignment is achieved—in part—*through* language itself (e.g., Casasanto & Lupyan, 2014; Dingemanse, 2017; Lupyan & Bergen, 2016). Learning and using a natural language may help people to not just convey, but also shape and structure their thoughts in ways that are—more or less—understandable to others. On this view, rather than just being a device for conveying our thoughts, language provides an interface between minds (e.g., Clark, 1998; Gentner & Goldin-Meadow, 2003; Gomila et al., 2012; Lupyan & Bergen, 2016). The current work is centered on testing a very specific (but strong) version of this hypothesis.

The idea that language may play a causal role in conceptual alignment is supported by several lines of evidence: some circumstantial, others more direct. First, there is the simple observation of enormous lexical diversity among languages (e.g., Enfield, 2004; Evans & Levinson, 2009; Wierzbicka, 2013). If our conceptual representations were naturally aligned—either due to shared biology, shared environment, shared learning mechanisms, or all three—and if words mapped onto these pre-existing conceptual representations, one would expect lexicons to show more similarity than they do (Thompson et al., 2020). And although it is clear that the lexical systems of natural languages occupy a small space of all possible systems (e.g., Kemp & Regier, 2012; Zaslavsky et al., 2018)—lexicons are hardly systems where “anything goes”—it is striking that finding universal units of linguistic meaning has been so difficult. Even in the domain of perception, where one might expect vocabularies to be most constrained by virtue of our shared biology, one finds tremendous diversity of naming schemes (Kay et al., 2011; Majid, 2021; Majid et al., 2018). Speakers within a language tend to align to a reasonable degree, for example, the hue boundary between blue and green is quite similar among English speakers (Forder & Lupyan, 2017), though even here, the extent of alignment depends on the measures one uses (Kuehni, 2004).

Second, prior computational and experimental work hints that the use of language can help align conceptual representations. For example, in a series of simulations with artificial agents, Steels and Belpaeme (2005) showed that allowing agents to label colors and then communicate those labels yielded much more similar systems of color categories than those that emerged in the absence of communication with labels. Although the environments and categorization constraints of the artificial agents were fixed, there still existed a wide variety of categorization solutions. Requiring agents to communicate narrowed the solution space resulting in more aligned categories. Using a very different approach, Roads and Love (2020) hypothesized that although unsupervised category learning from perceptual data is extremely difficult, it may be possible induce categories with surprising ease by aligning embedding spaces derived from multiple modalities (e.g., language, visual information, auditory information). They provide a compelling demonstration of such an alignment process. Critically, linguistic embeddings appear to be an especially important modality for this alignment process to work. To the extent that linguistic embeddings play a privileged role in this process, learners exposed to linguistic structure in addition to perceptual information should align their concepts much more effectively than in the absence of linguistic input.

On the experimental side, several studies have directly tested the role of language in conceptual alignment. Markman and Makin (1998) had pairs of participants build LEGO models with verbal coordination, e.g., “Then take the 2-dot white hinge with the hinges on the side” or without it. Participants who communicated during building subsequently showed more consistency in which pieces they treated as more and less similar (as revealed by a nonlinguistic sorting task) than participants who built the same LEGO models without verbal coordination. The authors concluded that “the act of establishing joint reference promotes consistency in people’s category structures” (p. 348). However, in addition to joint reference, the two conditions differed in whether they engaged in interactive communication and whether they performed a joint task. Suffill et al. (2016) showed that one can dispense with both interactive communication and the use of familiar labels. Participants tasked with sorting novel geometric shapes into two categories produced more similar sorts even though they never jointly interacted and even though the categories were labeled by nonsense words. In a subsequent study, Suffill et al. (2019) showed that (nonsense) labels also increased alignment for more meaningful stimuli (pictures of mountains), though only when participants had the goal to coordinate their categories.

Taken together, the simulations and experimental studies suggest that even in relatively simple domains such as shape and color categorization, the use of labels—even meaningless ones—can help constrain categorization schemes, leading to greater conceptual alignment. But how? And how much language does one really need?

### Current Studies

Here, we test a strong prediction of the claim that language—even in its most minimal form—can increase conceptual alignment. We test this prediction in a domain where perceptual and categorization constraints would seem to provide observers with all the information they need to produce highly aligned concepts: two-dimensional novel shapes (Experiment 1; Figure 1) and variants of familiar shapes (Experiment 2; Figure 8). We measured conceptual alignment by having participants arrange the shapes according to perceived similarity (Goldstone, 1994; Malt et al., 1999): the more similar the sorts between two people, the more aligned we took them to be. Importantly, the stimuli in both experiments could be easily clustered into two perceptually distinct categories based on visual properties alone. We compared sorting under three conditions: A label condition where people were exposed to incidental nonsense labels for the two shape categories, a no-label condition where people were exposed to the category structure of the stimuli but without category names, and a baseline condition in which people sorted the shapes without any prior exposure to the shapes or categories. These three conditions allowed us to compare how conceptual alignment is affected by verbal labels as compared to shared perceptual experience alone.

**Figure 1.**
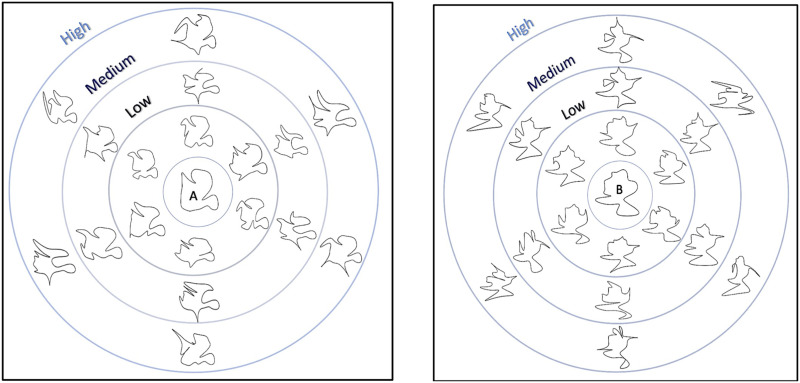
Category A (left) and B (right) prototypes and exemplars, showing the “low”, “medium” and “high” distortions.

In addition to testing whether language increases conceptual alignment even in these very strict conditions (minimal language, no interaction, perceptually “obvious” categories), our analyses allowed us to distinguish between two sources of observed differences in alignment:

(1)  *Labels as guides to the number of categories*. Category names can be a useful guide to the *number* of categories the learner should form (Plunkett et al., 2008). For example, suppose a learner encounters various four-wheeled vehicles. How should these vehicles be categorized? The number of labels one encounters can be a guide to the level of desired granularity (Bloom, 2002). Encountering two labels may lead the learner to group them into, say, cars and trucks. Encountering three may encourage a further subdivision of cars into coupes and sedans. Two learners exposed to the same number of labels would be more likely to form the same number of categories which, all else equal, would lead to greater conceptual alignment than if different learners assumed different numbers of categories.
(2)  *Labels as reifiers of categories*. Labels can promote alignment even when the number of desired categories is not in question. Previous work has shown that even when learners knew that there were precisely two categories (i.e., two types of “aliens”, one of which had to be approached and one escaped from), exposure to redundant labels helped learners learn which ones are which (Lupyan et al., 2007). Associating labels with various specific exemplars that comprise the category allow learners to more efficiently home in on the features that are most diagnostic of category membership (James, 1890; Lupyan, 2012b). Subsequent work provided converging evidence that labels help learners identify category-relevant features (Althaus & Mareschal, 2014; Barnhart et al., 2018). On this account, exposure to labels may lead learners to represent the labeled objects in a more categorical way (Lupyan & Thompson-Schill, 2012), sensitizing them to features most diagnostic of the categories. This view leads to several predictions: (a) Exposure to labels should lead to more categorical sorts as evidenced by reduced within-category distances and/or increased between-category distances. (b) Forming representations that are more categorical should be associated with greater alignment. Participants with more categorical sorts should align better with one another than participants with less categorical sorts.^2^ (c) If greater alignment from labels is caused by greater categoricality, then categoricality should mediate the relationship between alignment and labels such that controlling for categoricality should reduce or eliminate differences in alignment between label and no-label conditions.

## EXPERIMENT 1

### Methods

#### Participants.

We obtained full data from 129 (85 female; 43 male) psychology students (Ages 18–22, M = 18.8, SD = 0.7 years) from University of Wisconsin-Madison. Participants were randomly assigned to a *Baseline* (N = 45), *No Labels* (N = 43) or *With Labels* (N = 41) condition. We excluded an additional 39 participants who failed to move all items during the free sort phase. We subsequently clarified the instructions and modified the experiment code to ensure that all items are moved during the sort.

#### Stimuli.

We constructed two family-resemblance type categories designed to be difficult to name, but easy to distinguish (see Figure 1)^3^. We began by manually creating shapes defined by 39 points (vertices) and connected them with an interpolated spline. From these we used (informal) pilot-testing to select two shapes that were both difficult to name and easy to distinguish from one another. These served as the two prototypes (Figure 1-top). We then generated 18 unique exemplars per category by perturbing the prototype vertices and fitting new splines thus creating low, medium, and high distortions (see Figure 1-bottom for several examples). The perturbation was achieved by adding Gaussian noise (mean = 0, SD as described below) to the x and y coordinates of the vertices (each fifth vertex to maintain smoothness) and then re-interpolating the spline. In Experiment 1, we created exemplars at three different levels of noise: SD_low_ = .25, SD_medium_ = .55, SD_high_ = .75.

For the label condition, each category was paired with an auditory label—the nonsense words “talp” and “gek” recorded by an American English speaker. The assignment of labels to categories (A vs. B) was counterbalanced. To equate auditory exposure, participants in the “No Labels” condition heard white noise length in place of the labels. The white noise was matched for length and volume to the spoken labels (e.g., as in Forder & Lupyan, 2019; Lupyan, 2008b).

### Procedure

The experiment was programmed in PsychoPy (Peirce et al., 2019). Participants assigned to the “Label” or “No-Label” conditions began with a Pre-exposure phase before proceeding to the free-sort phase. Participants assigned to the “Baseline” condition proceeded directly to the free-sort phase.

#### Pre-exposure.

This phase had three purposes: First, to familiarize participants with the visual stimuli. Second, to give than opportunity to explicitly contrast the stimuli from the two categories in a controlled way. Third, to incidentally expose participants in the *With Labels* condition to the two category labels.

We used an xAB match-to-sample task (Figure 2). On each trial, participants saw a random shape presented at the top of screen. This shape (the *x* of the xAB) then disappeared and, after a 1s delay, participants saw two shapes (the AB of the xAB) and had to indicate which of these exactly matched the top shape. In the *With Labels* condition, the top shape was presented alongside an auditory label “a talp” or “a gek” depending on its category. In the *No Labels* condition, the top shape was accompany by a white noise instead. Importantly, the A and B options were always from different categories, ensuring that participants in both the *With Labels* and *No Labels* conditions had equal experience making between-category comparisons. The display with the two choices remained visible until a response was made. The correct response was counterbalanced across left and right positions. Errors were signaled with a buzzing sound.

**Figure 2.**
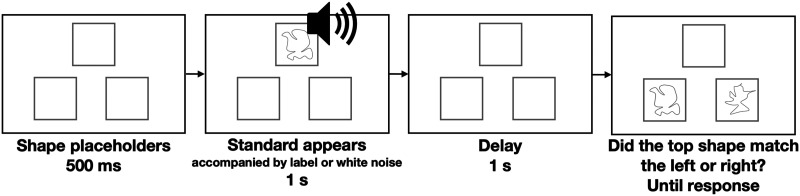
Schematic of delayed match-to-sample task used for the pre-exposure. Participants responded with which shape matched the sample. In the *With Labels* condition, the top shape was accompanied by an auditory category label. In the *No Labels* condition, the label was replaced by white noise.

There were a total of 243 trials: 3 blocks 81 trials comprising 9 shapes from each of the two categories, paired with 9 shapes from the other category. Category prototypes were not presented during this part of the experiment.

#### Free Sort.

Participants were shown 20 shapes: 10 A category shapes and 10 B category shapes. These shapes included 3 previously seen exemplars, 6 previously-unseen exemplars at different levels of distortion, and the two category prototypes (also previously unseen). The shapes were initially positioned around the perimeter of the screen, and participants were asked to drag the shapes and arrange them based on similarity, forming any number of clusters they wished (Figure 3). Shapes were allowed to overlap. Partway through the experiment, we added the instruction that participants had to move all shapes to complete the sort, ensuring that the final position of a shape was not simply its random starting location.

**Figure 3.**
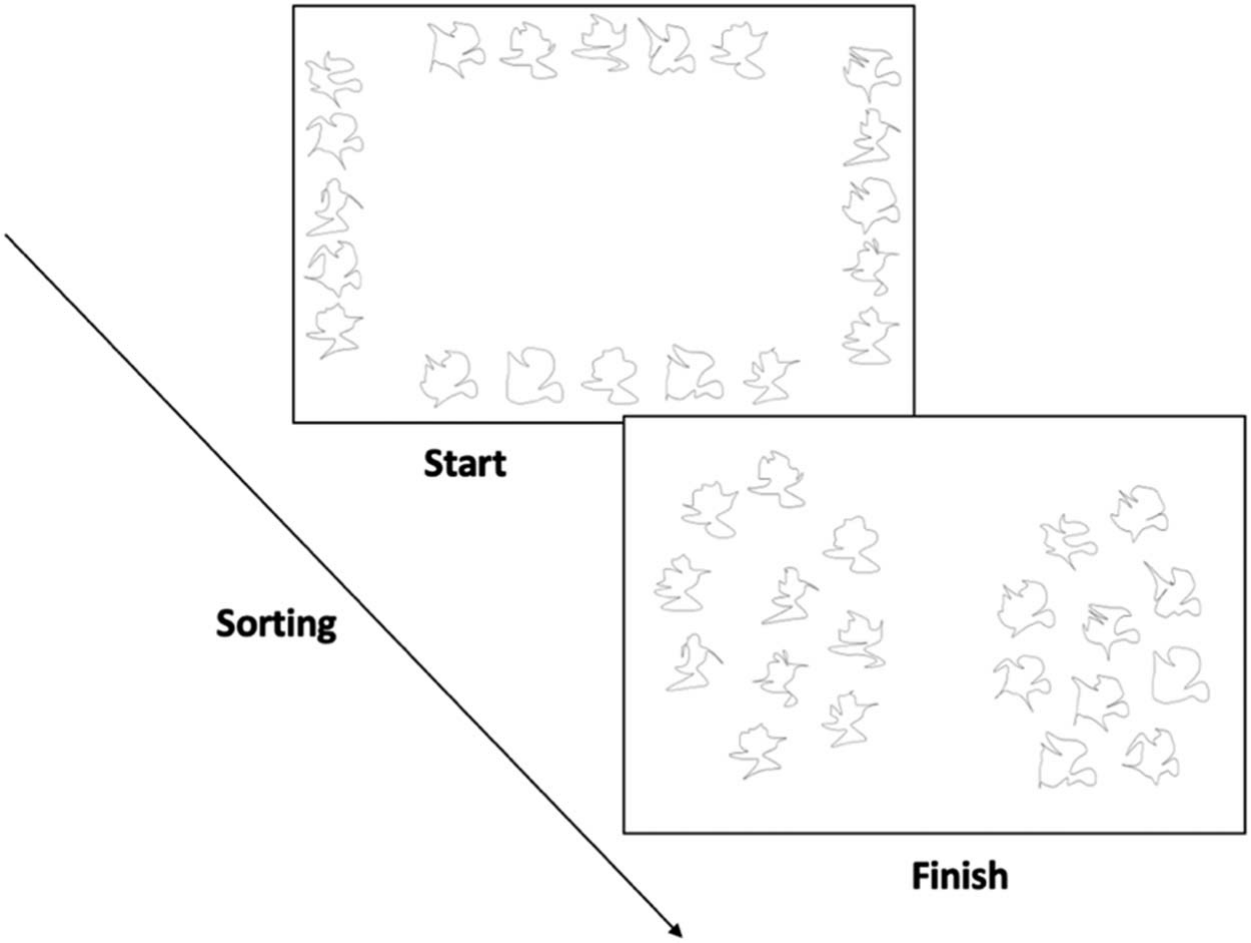
The free-sort task in its initial state (left) and an example of a finished sort (right).

### General Analytic Approach

Raw data and analyses are available at https://osf.io/qc94m/. We used mixed effects linear regression for continuous dependent variables and mixed effects logistic regression for discrete variables, as implemented in R’s lme4 package v. 1.1.30 (Bates et al., 2014). Predictors were centered. Models included by-subject random intercepts and random-slopes for within-condition factors unless adding these prevented convergence.

### Specific Hypotheses and Operationalization of Key Measures

Recall that we designed the stimuli to be easy to group into categories based simply on their visual appearance. Finding categorical structure in the sorts produced by participants in the *Baseline* condition would confirm that this categorical structure was indeed readily perceived. The more interesting comparisons involve the two groups who had identical pre-exposure to the shapes, differing only in exposure to labels. In turn, we tested the following hypotheses: (1) *With label* participants will produce more categorical sorts; (2) *With label* participants produce sorts with fewer clusters (and specifically containing two clusters); (3) *With label* participants will be more aligned with one another. We then investigated the relationship between number-of-clusters, categoricality, and alignment to determine whether any changes in alignment caused by exposure to labels were better explained by labels guiding participants to the same number of categories or by labels making people’s representations more categorical.

We operationalized *categoricality* of each person’s sort as the ratio: M_between-distance_ / M_within-distance_. The mean between-distance was the Euclidean distance in pixels between all the pairs of between-category shapes, e.g., the distance between item A1 and B1, A1 and B2, etc. Mean within-distance was computed in the same way except taking just the same-category pairs, e.g., the distance between A1 and A2, B1 and B2, etc. We then log-transformed the ratio to achieve a more normal distribution. This way of computing categoricality (in comparison to simply subtracting the distances) is more robust to differences between participants using more or less of the canvas area to perform their sort.

#### Number of Clusters.

We grouped each person’s final item locations into medoid-based clusters using the “pamk” function in R (Hennig, 2023). A medoid was defined as the item within a cluster for which the average distance between it and all other cluster members is smallest (Kaufman & Rousseeuw, 2009). We statistically compared the number of clusters participants formed using Poisson regression.

#### Alignment.

To quantify alignment between two people’s sorts, we used the following procedure: For each participant, we took the Euclidean distance between all the item pairs for their sort and computed the (Fisher’s z-transformed) rank correlations between that participant’s pairwise item distances and the pairwise item distances of the other participants in the same condition. All items were treated identically meaning that our alignment measure was “blind” to the category identity of the items.

Using participant dyads as units of measurement leads to the problem that some dyads are non-independent, e.g., the alignment between subject_1_ and subject_2_ is more related to the alignment between subject_1_ and subject_3_ than between subject_3_ and subject_4_. We therefore computed the alignment between each subject and all the other subjects from the same condition, and used standard linear models with the participants’ averaged alignment as the outcome variable. In the Appendix, we supplement this result with a more conservative analysis using *lmerMultiMember* (van Paridon et al., 2022), an R package that allows for specifying multiple membership random effects and attribute the variance associated with both individuals comprising each dyad from non-aggregated data. The Appendix also includes Example sorts of relatively high, medium, and low alignment.

#### Bridging Individual and Group Measures.

Our analyses of participants’ sorts use measures defined for individuals (categoricality, number of clusters) and alignment, a measure only defined for a group (here, participant dyads). To determine the relationship between the two types of measures requires deciding how to translate the individual measure to a group setting. For example, suppose the sort of participant 1 has a categoricality value of 1, the sort of participant 2 has a categoricality value of 0.4, and that the alignment between participant 1 and 2 is 0.3. Which categoricality value do we use if we want to predict alignment from categoricality? We explored several methods to produce a single value per participant-dyad: using the minimum value (here, 0.4), the maximum value (here, 1), and the absolute difference (here, 0.6).

### Results

#### Pre-exposure.

Accuracy on the delayed match-to-sample task was high and nearly identical for the *No Labels* (*M* = 0.981, SD = 0.02) and *With Labels* groups (*M* = 0.982, SD = 0.02), *z* < 1, confirming the ease of the match-to-sample task and the high discriminability of the two categories. Before analyzing Reaction Times (RTs) for correct judgments, we excluded RTs < 150 ms. or >2 SDs of the subject’s mean. RTs were faster for the *No Labels* condition (*M* = 585 ms., *SD* = 91 ms.) compared to the *With Labels* condition (*M* = 692 ms., *SD* = 167 ms.), *b* = 107, *SE* = 29, *t* = 3.66, *p* < .001.

#### Free Sort.

We next examined how participants sorted the shapes.

#### Categoricality.

**Figure 4.**
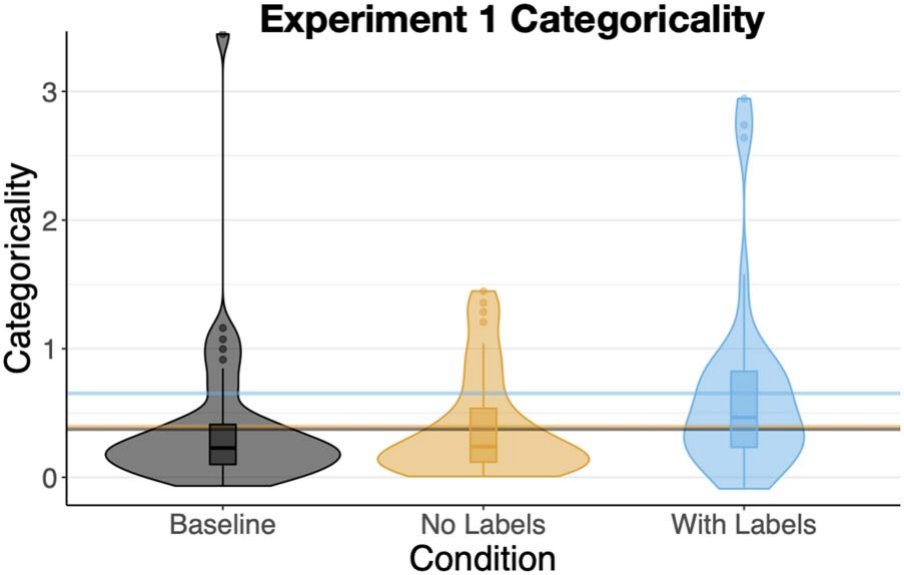
Mean Categoricality for Experiment 1. Vertical lines show condition means.

Positive categoricality scores indicate that participants placed within-category items closer together than between-category items (because log[≥1] is ≥0). That is, A’s were grouped with other A’s and B’s with other B’s. Note that a participant who formed tight clusters composed of a mix of A’s and B’s would have low categoricality on this measure. As shown in Figure 4, participants in all conditions had positive categoricality scores indicating that even without pre-training, participants’ sorts reflected the visual category structure we built into the stimuli. However, sorts by the *With Labels* participants (M = .65, SD = .70) were significantly more categorical than sorts by participants in both the *Baseline* condition (M = .38, SD = .56), b = .28, SE = .12, t = 2.3, p = .024, and also more categorical than sorts of those in the *No Labels* condition (M = .40, SD = .40), b = .26, SE = .12, t = 2.1, p = .038. Categoricality in the *Baseline* and *No-Labels* conditions was nearly identical, t < 1, showing that prior exposure to the shapes in a categorical context without labels on its own did not increase categoricality.

#### Number of Clusters.

**Figure 5.**
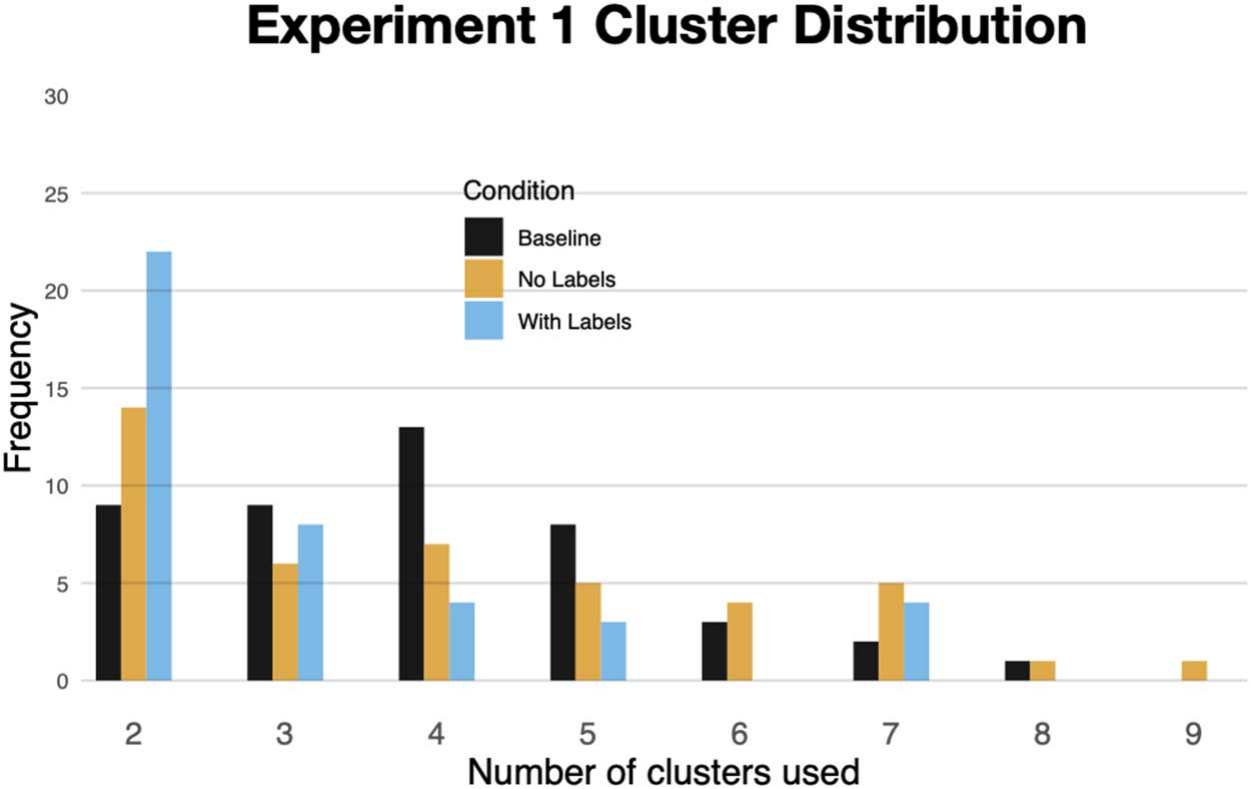
The number of clusters formed during free-sort in Experiment 1.

Participants in the *With Labels* condition produced sorts containing an average of 3.1 clusters, significantly fewer than participants in the other two conditions: *No Labels* (M = 4.1), z = 2.34, p = .04; *Baseline* (M = 3.9), z = 2.34, p = .02. Figure 5 shows the distribution of sorts. As the figure makes clear, exposure to labels led to a higher proportion of 2-cluster sorts (M = 53%). This proportion was significantly higher than 2-cluster sorts in the *Baseline* condition (M = 20%), z = 3.14, p = .002, and marginally higher than 2-cluster sorts in *No Labels* condition (M = 33%), z = 1.93, p = .05.

#### Alignment.

**Figure 6.**
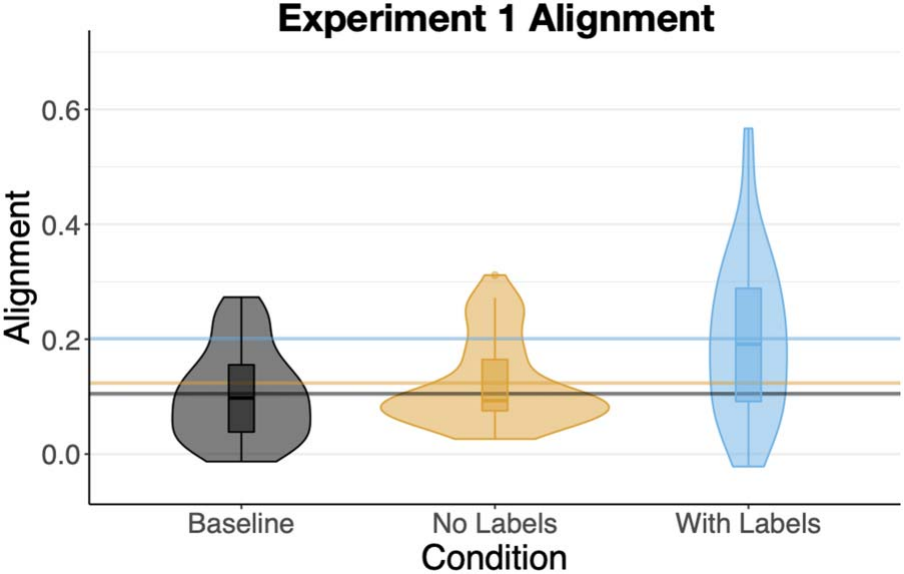
Mean alignments of sorts in Experiment 1.

The sorts of participants in the *With Labels* condition (M = .20, SD = .14) were significantly more aligned than the sorts in the *Baseline* condition (M = .11, SD = .08), b = .07, SE = .02, t = 3.55, *p* < .001 and also more aligned than the sorts of participants in the *No Labels* condition (M = .12, SD = .07), b = .10, SE = .02, t = 4.45, *p* < .001 (Figure 6). Alignment in the *Baseline* and *No Labels* conditions was nearly identical, t < 1.

Next, we examined whether differences in alignment were mediated by differences in categoricality and the number of formed clusters. Alignment was correlated with all three types of pairwise cluster measures, but to different degrees: There was a very high correlation between alignment and *minimum* pairwise categoricality was r = .92, *p* < .0001, followed by the *maximum* pairwise categoricality, r = .77, *p* < .0001, and *absolute difference* in categoricality values, r = .60, *p* < .0001. This pattern suggests that the participant with the less categorical sort acts as a lower bound on pairwise alignment. We use the *minimum* pairwise categoricality in the analyses below.

**Figure 7.**
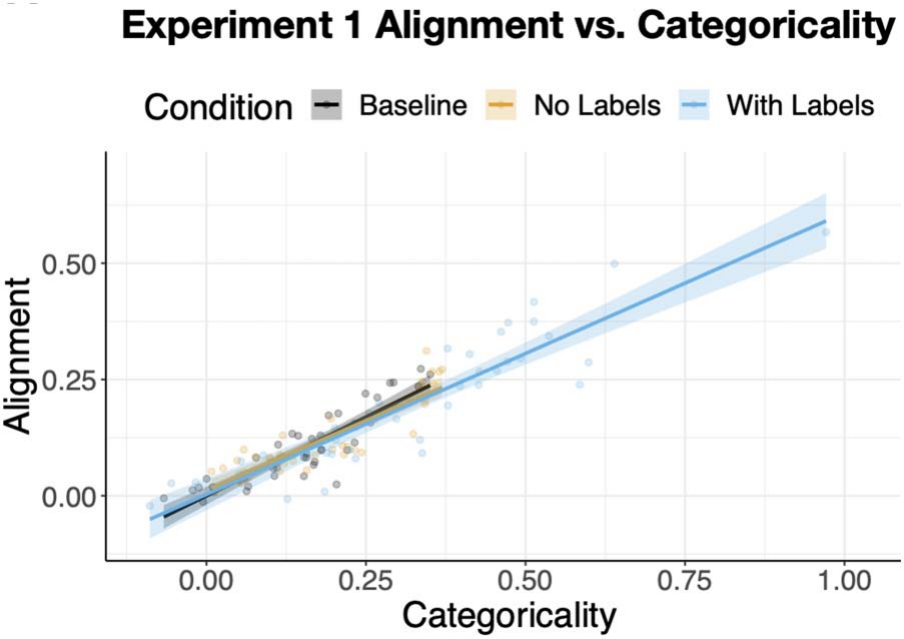
The relationship between categoricality and alignment in Experiment 1. Each dot represents an individual participant with their mean alignment to the remaining participants in their condition, and the mean of the minimum pairwise categoricality scores. Categoricality fully mediated the effect of condition on alignment.

Entering categoricality as a covariate revealed that it *completely* mediated the effect of condition on alignment, accounting for 84% of the variance in alignment: b = .62, SE = .026, t = 23.38, *p* < .0001 (Figure 7). That is, controlling for categoricality, the effect of condition on alignment was essentially 0: b = .009, *p* > .3. In contrast, the number of clusters participants formed was much less related to alignment. Dyads with a higher minimum number of clusters were significantly less aligned, r = −.26, p = .003. So were dyads with higher maximum number of clusters, r = −.22, p = .01. Absolute difference in the number of clusters was not associated with alignment, *p* > .4. Critically, including the number of clusters as a covariate when predicting alignment from condition showed that the relationship was unchanged (i.e., there was no mediation) and furthermore that the inclusion of condition eliminated the relationship between alignment and cluster number reported above, p’s > .2.

### Discussion

Even brief exposure to category labels—the nonsense words “talp” and “gek”—was sufficient to increase categoricality of the produced sorts. In turn, more categorical sorts were associated with greater alignment. This result is all the more surprising because participants in the *Baseline* condition still grouped within-category items closer to one another than between-category items, i.e., they appreciated the categorical structure of the stimuli. Yet exposure to (nonsense) labels nevertheless increased alignment.

Category labels did encourage participants to form fewer clusters; they were somewhat more likely to form exactly two. However, this cluster number had little effect on alignment. What mattered was *categoricality*. The results are consistent with the prediction that labels can increase alignment even when we hold constant their role as *guides* to how many categories should be formed. Remarkably, the finding of higher alignment in the *With Labels* compared to the *No Labels* condition persisted even when we compared *only* the participants who produced 2-cluster sorts. The finding of a very strong (r > .9) relationship between categoricality and alignment and that the difference in alignment between conditions was eliminated when we controlled for categoricality both support the claim that labels can reify categories rather than just serving as guides to category granularity.

One aspect of the results gives us pause: participants in the *With Labels* condition spent slightly, but significantly, more time on the match-to-sample task than those in the *No Labels* condition, presenting a potential confound. In Experiment 2, we conducted an even stronger test of the hypothesis that exposure to labels increases conceptual alignment via categoricality using a design that we thought would eliminate this RT difference in the match-to-sample task.

## EXPERIMENT 2

In Experiment 1, we deliberately used unfamiliar and difficult-to-name shapes to avoid participants in the *Baseline* and *No Labels* conditions from generating their own labels during the sort. In Experiment 2 we did away with this feature of the design. Rather than using irregular shapes as category prototypes, we seeded the two categories with familiar and nameable shapes: a circle and a square. This change allows for a still stronger test of the aligning power of category labels. The familiarity of the prototypes should, in principle, allow participants to sort according to the familiar categories *circle* and *square*. Hearing unfamiliar labels paired with familiar categories could even interfere with people’s ability to use these categories as a basis for sorting. Nevertheless, we hypothesized that explicit category labels (even nonsense ones) would lead participants to form more categorical representations, possibly by helping to either extract stably represent category-diagnostic features (Lupyan, 2008a, 2012a). Other changes included recruiting a more diverse group of participants, testing them in a less-controlled online setting, and using a briefer pre-exposure phase. Finding that labels continue to increase alignment even under these more stringent conditions would lend further support to the idea of label-induced conceptual alignment.

### Methods

#### Participants.

We obtained full data from 122 participants (55 female, 65 male, 2 other), all recruited online. Some were psychology students participating for credit, others were recruited through Amazon Mechanical Turk and participating for payment. Ages: 18–70 years (*M* = 34.7, *SD* = 13.7)^4^. Participants were randomly assigned to the *Baseline* (*N* = 41), *No Labels* (*N* = 37) and *With Labels* (*N* = 44) conditions. We excluded an additional 65 participants: Of those who participated in the pre-exposure task, 19 participants scored less than 80% accuracy on the match-to-sample task (< 80% accuracy) and 8 participants scored less than 7/9 correct on catch trials (see below). Thirty-eight participants failed to complete the sorting task, e.g., many just nudged all the shapes from their starting positions which allowed them to submit the task without making a good-faith sorting attempt.

#### Stimuli.

**Figure 8.**
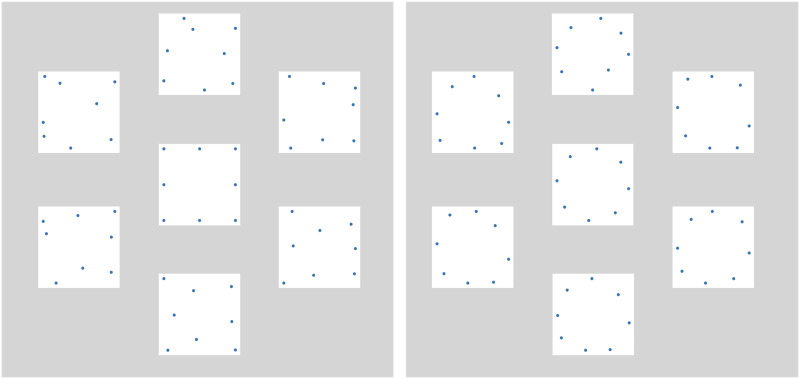
Samples of stimuli used for Experiment 2 based on a “circle” (left) and “square” (right) prototypes. The central prototypes were only shown in the sorting phase.

In Experiment 2, the same stimuli generation procedure was used as in Experiment 1, except the stimuli were the points themselves with no contour connecting them (Figure 8). The individual exemplars were generated by perturbing the coordinates of the vertices by adding Gaussian noise to the coordinates, but now at the same level (SD = .35). We generated 10 exemplar per category. The labels were the same as in Experiment 1. Participants in the *No Labels* condition again heard white noise in place of the spoken labels. All tasks were administered through a web browser using jsPsych (de Leeuw, 2015).

### Procedure

#### Pre-exposure.

We used the same match-to-sample task as in Experiment 1, but with fewer trials to accommodate the relatively less patient online participants (108 trials vs. 243 used in Experiment 1). We also included 9 catch trials to check that participants were paying attention to the audio: participants would be asked to listen and type out a spoken word (e.g., ‘apple’, ‘bike’) at random points during the task.

#### Free Sort.

The free sort procedure was identical to that in Experiment 1. Participants were required to move all the items from their starting locations before they could submit the task.

### Results

#### Pre-exposure

##### Average Accuracy.

Average accuracy on the delayed match-to-sample task was nearly identical: *M* = 0.97 (*SD* = 0.02) for the *No Labels* condition and *M* = 0.97 (*SD* = 0.02) for the *With Labels* condition, z < 1.

##### Average Reaction Time.

As expected given the greater visual confusability of the exemplars compared to those of Experiment 1, RTs were significantly longer than in Experiment 1, b = 134, SE = 25.03, t = 5.35, *p* < .0001. Unlike Experiment 1, average reaction times for correct responses was nearly identical in *No Labels* condition (*M* = 773 ms., *SD* = 179 ms.) and “With Labels” (*M* = 769 ms., *SD* = 179 ms.), *t* < 1. If we continue to see differences in alignment, they could not have come from a difference in inspection times during the match-to-sample task.

#### Free Sort

##### Categoricality.

**Figure 9.**
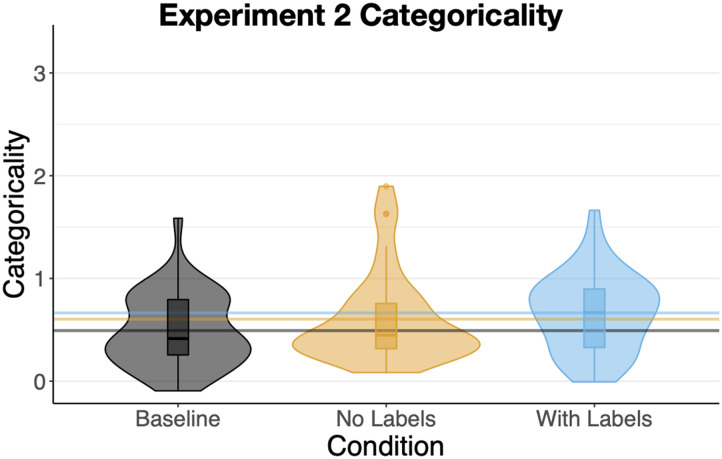
Mean Categoricality for Experiment 2. Vertical lines show condition means.

As in Experiment 1, participants in all conditions had positive categoricality scores (Figure 9). *Baseline* categoricality (M = .49, SD = .36) was significantly lower than categoricality in the *With Labels* condition (M = .66, SD = .38), b = .17, SE = .08, t = 2.03, p = .04. Categoricality in the *With Labels* condition was numerically, but not significantly greater than in the *No Labels* condition (M = .60, SD = .44), t < 1. There was no difference in categoricality between the *No Labels* and *Baseline* conditions, *p* > .2. Overall categoricality was marginally greater compared to Experiment 1, b = .12, SE = .06, t = 1.89, p = .06 consistent with participants being more reliant on their pre-existing knowledge of circles and squares.

##### Number of Clusters.

**Figure 10.**
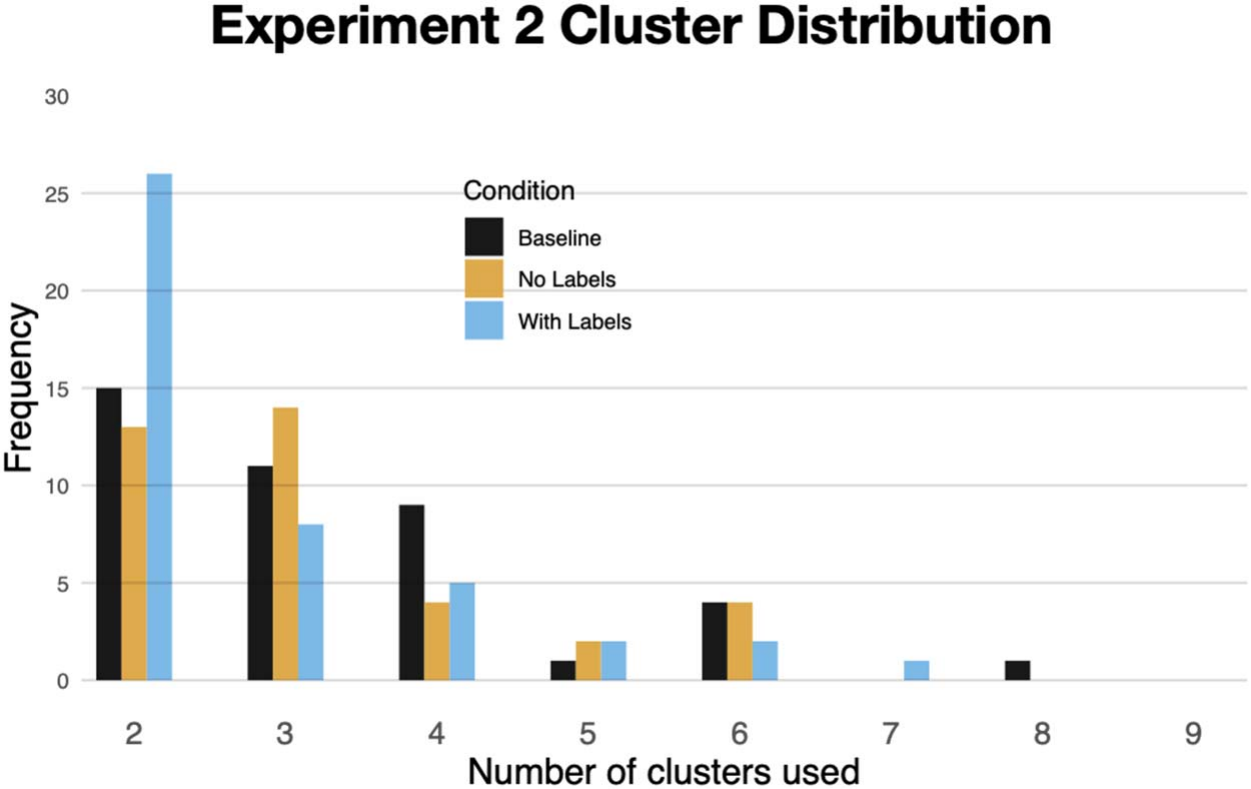
The number of clusters formed during free-sort in Experiment 2.

Participants in the *With Labels* condition produced sorts containing an average of 2.8 clusters, which was not significantly different from participants in the *No Labels* condition (M = 3.2), z < 1, and *Baseline* (M = 3.31), z = 1.25, p = .21. Figure 10 shows the distribution of sorts. Despite the lack of overall difference, those in the *With Labels* condition were significantly more likely to produce 2-cluster sorts (M = 60%) than those in the *Baseline* condition (M = 37%), b = .92, SE = .44, t = 2.06, p = .04, as well as those in the *No Labels* condition (M = 35%), b = .98, SE = .46, t = 2.12, p = .03.

##### Alignment.

The sorts of participants in the *With Labels* condition (M = .35, SD = .17) were significantly more aligned than those in the *Baseline* condition (M = .21, SD = .12), b = .14, SE = .03, t = 4.63, *p* < .001 and also more aligned than the participants in the *No Labels* condition (M = .27, SD = .11), b = .07, SE = .03, t = 2.37, p = .02 (Figure 6B). Unlike Experiment 1, alignment in the *No Labels* condition was significantly greater than in the *Baseline* condition, b = .07, SE = .03, t = 2.10, p = .04. Overall alignment in Experiment 2 was considerably higher than in Experiment 1, b = .13, SE = .02, t = 8.31, *p* < .0001, again consistent with the possibility of participants relying on their knowledge of circles and squares. The increase in alignment between Experiment 1 and 2 was numerically smaller in the *Baseline* condition (M = .10) compared to the other two (M = .15) though the interaction was not significant.

We next examined whether, as in Experiment 1, differences in alignment were mediated by differences in categoricality and the number of clusters. The *minimum* pairwise alignment was again much more strongly related to alignment, r = .94, *p* < .0001, than maximum pairwise alignment: r = .71, *p* < .0001, and absolute-difference in pairwise categoricality, r = .13, p = .16.

When entered as a covariate in a model predicting alignment from condition, minimum pairwise categoricality accounted for 89% of the variance (Figure 12), b = .78, SE = .027, t = 29.23, *p* < .0001. The difference in alignment between *Baseline and With Labels* was now eliminated, *p* > .2, but a smaller yet significant alignment advantage of *With Labels* compared to *No Labels* remained, b = .02, SE = .01, t = 2.3, p = .02. In sum, whereas categoricality completely mediated the relationship between condition and alignment in Experiment 1, here we saw partial mediation.

Dyads with a higher minimum number of clusters were significantly less aligned, r = −.37, *p* < .001. So were dyads with higher maximum number of clusters, r = −.42, *p* < .001 as well as dyads with a larger absolute difference in the number of clusters, r = −.36, *p* < .001. As in Experiment 1, including the number of clusters as a covariate when predicting alignment from condition left the relationship unchanged (i.e., there was no mediation). Regardless of which of the three measures of cluster number was used, categoricality continued to be an extremely strong predictor of alignment (t > 20).

### Discussion

**Figure 11.**
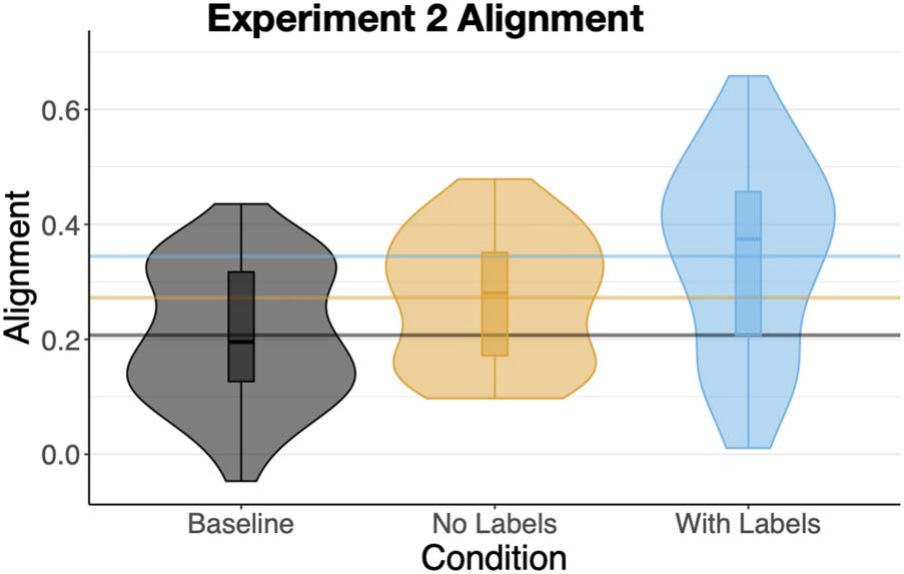
Mean alignments of sorts in Experiment 2.

One might imagine that when faced with perturbations of circles and squares (Figure 8) participants in all three conditions would simply sort them into two clusters: one containing the more circular shapes and the other more-square-like ones. Indeed, many did exactly this. However, brief exposure (now even briefer) to the items in a context encouraging more categorical processing led to an increase in categoricality (Figure 9), two-cluster sorts (Figure 10), and alignment (Figure 11). Exposure to explicit category labels—the nonsense words “gek” and “talp”—further increased alignment, as in Experiment 1. And, as in Experiment 1, the increased alignment was mediated by an increase in categoricality, but not by an increase in the number of clusters—consistent with the idea of labels reifying categories rather than just informing learners about the desired level of category granularity.

**Figure 12.**
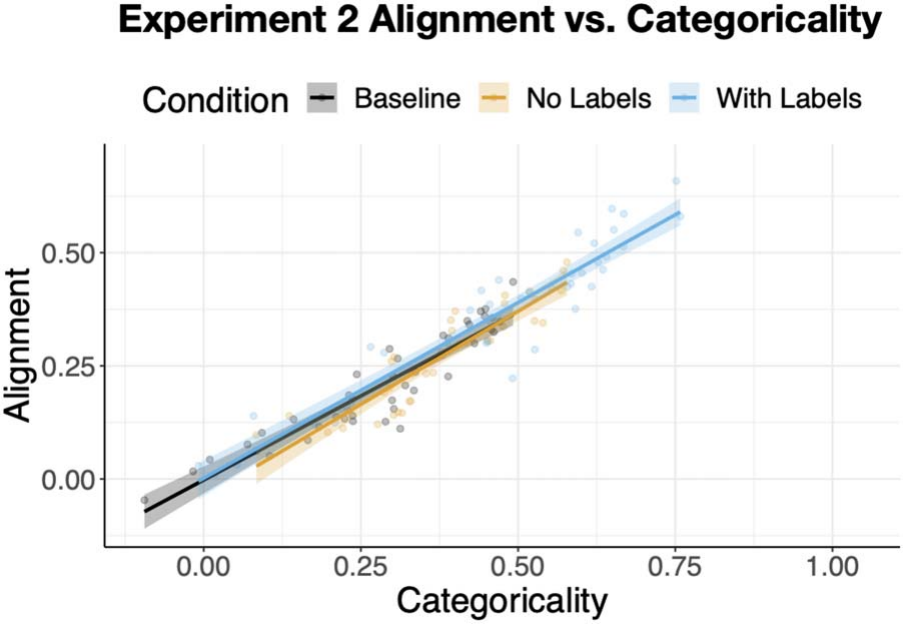
The relationship between categoricality and alignment in Experiment 2. Categoricality partially mediates the effect of condition on alignment. Each dot represents an individual participant with their mean alignment to the remaining participants in their condition and the mean of the minimum pairwise categoricality scores.

## GENERAL DISCUSSION

Successful use of language requires conversants to be at least somewhat conceptually aligned. Might language itself have something to do with it by helping induce more similar conceptual representations than people would have in the absence of shared linguistic experience? The present study provides a limited, but strong test of the hypothesis that even a very minimal form of language—nonsense and redundant category labels in the absence of communication—can increase conceptual alignment over and above shared perceptual experiences alone.

Prior work has shown that labels can increase representational alignment even in the absence of their use in communication (Suffill et al., 2016, 2019). The present study provides a more thorough examination of label-induced alignment by testing the hypothesis that labels increase alignment by promoting more categorical representations of items. This idea stems from Lupyan’s label-feedback hypothesis according to which associating a label with multiple exemplars leads to it being associated with category-diagnostic properties because those links receive the most reinforcement from one encounter to the next (Lupyan, 2012a; see also Forder & Lupyan, 2019; Lupyan, 2008a; Lupyan & Thompson-Schill, 2012). Subsequent presentation of the items activate the category label, which, through a process of feedback, activates (or helps keep active) those category-diagnostic properties thereby creating a more categorical representation of the item (see Lupyan, 2012b for a model). Greater alignment is one consequence of increased categoricality because as items become more categorical, they become more typical and their representations tend to converge. Consider for example the greater similarity between two typical letter As (A and A) compared to less typical ones (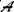 and 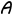). In addition to increasing categoricality, exposure to the two category labels also encourage people to form fewer and/or exactly two clusters. But although dyads with different numbers of clusters were, on average, less aligned, number of clusters did not mediate the relationship between alignment and condition. In contrast, categoricality did (fully in Experiment 1, partially in Experiment 2). Taken together these results suggest that labels do not merely guide learners to the desired number of categories, but *reify* the categories—leading to more categorical and hence more aligned representations.

Although our alignment measure was “blind” to the category status of the items, the very high correlation between pairwise alignment and categoricality raises the question of whether it is somehow arithmetically inevitable^5^. To address this question, we simulated participants placing items on a canvas using different strategies and then computed categoricality and alignment between their sorts. If item placement is purely random, there is no relationship (r ∼ 0) between categoricality and alignment (See Figure 1S in the Appendix). Of course, purely random sorts are exceedingly unlikely to result in alignment. A simulation of 3000 participants produced a maximum alignment of just .06. High alignment in the absence of any clusters is clearly possible (e.g., two participants can, in theory, sort the items into evenly-spaced grid (Figure S2) which can perfectly align in the absence of any clusters whatsoever. However, in practice, this never happens. In practice, high alignment comes from two participants clustering items in the same way. In theory, two participants could increase their alignment by forming clusters that mix items from A and B categories in the same way (e.g., if both sorts contained a cluster with items A^1^, B^1^, A^2^, and B^2^)—high alignment with low categoricality (as we defined it here). In reality, this did not happen. In virtually all cases, high alignment stemmed from participants sorting A’s with other A’s and B’s with other B’s. The Appendix also includes additional analyses showing how alignment differs across conditions when only within-category item distances are included (a measure of how similarly participants sorted items *within* each category) as well as alignment based on just between-category comparisons.

Our studies were not designed to compare the effects of verbal labels to other kinds of overt category markers and should not be interpreted to mean that verbal labels are the only or the best way to achieve increased alignment. A further limitation of our design, is that our With Labels and No-Labels conditions differed not only in the use of labels, but also in the use of language more generally (recall that the No Label condition used a bit of white noise in place of the phrases “a gek” and “a talp”). Although the white noise equates auditory exposure in the two conditions, it is conceivable that its presentation alongside items from the two categories led participants to unify them into a single category. If so, the differences between the With Labels and No-Labels conditions may have more to do with white noise unifying otherwise distinct stimuli rather than the labels separating them. We think this is unlikely for three reasons. First, in most analyses, it was the Label condition that was different from the other two, an unexpected pattern if the white noise worked to unify the stimuli into a single category. Second, not a single participant in any of the conditions formed a one-cluster sort as might be expected from someone who unified the stimuli into a single overarching category. Third, the results in Experiments 1 and 2 are essentially the same even though the category structure in Experiment 2 is even more apparent.

A related concern is that the white noise may have led participants to pay less attention to the stimuli in the pre-exposure phrase compared to the Label condition such that the results we are seeing in the Label condition may have come about from language in general and would have been the same even without explicit labeling of the two categories. Speaking against this interpretation is our finding that participants in the *No Labels* condition in Experiment 1 performed *faster* on the pre-exposure match-to-sample task compared to the *With Labels* condition—unexpected if they were paying less attention or were otherwise less engaged (one of the goals of Experiment 2 was to eliminate this difference in performance, which we did while replicating our key findings).

We tested the effects of labels on alignment in a highly constrained space, using only two categories. This is obviously a gross simplification of reality. Categories in the real world are much more numerous, and enter into complex causal and hierarchical relationships, none of which were investigated here. While limiting the generalizability of our results, our finding that exposure to labels promotes alignment even in this very minimal case suggests interesting avenues for future investigations both in children and children. Consider the classic study by Gelman and Markman (1986) in which they investigated whether preschool-aged children’s inferences about unseen properties was guided by category membership or visual similarity. In a typical trial, a child was shown a picture of a squirrel and told that it eats bugs, followed by a picture of a rabbit that “eats grass”. The child was then shown another animal—a kaibab squirrel that looks more like a rabbit than a conventional squirrel—and asked whether “this squirrel” eats grass or bugs. Preschool-aged children, much like adults, tended to favor the categorical match over the perceptual match. There is little reason to think that the *capacity* for such category-based induction is linguistic in origin, but it is telling that the tested children required an experimenter to explicitly use a verbal label to denote the categorical relationship^6^. In studies of adult categorical induction (e.g., Osherson et al., 1990), the categories over which inferences are being performed (e.g., mammals) are not explicitly provided, but the categorical response patterns may nevertheless rely on participants using such labels either implicitly or explicitly. It remains an open question whether their use promotes greater alignment in this domain.

Our finding that verbal labels increase conceptual alignment for simple shapes is just one result using specific stimuli and a particular way of measuring alignment. Our hope is that future investigations can map out the generality of the links between labels, categorization, and alignment, helping to inform future studies of both human-human and human-machine alignment (Rane et al., 2024).

## FUNDING INFORMATION

This work was partially supported by NSF-PAC #2020969 to G. L.

## AUTHOR CONTRIBUTIONS

E.S.: Conceptualization; Data curation; Formal analysis; Visualization; Writing – original draft; Writing – review & editing. J.v.P.: Formal analysis. G.L.: Conceptualization; Formal analysis; Visualization; Writing – original draft; Writing – review & editing.

## DATA AVAILABILITY STATEMENT

Data and analyses are available at https://osf.io/qc94m/.

## Notes

^1^ We use “concepts” and “word meanings” somewhat interchangeably, following Casasanto and Lupyan (2014), but oftentimes they can and should be distinguished (Levinson, 1997; Lupyan, 2012a; Wolff & Holmes, 2011). The relationship between them is asymmetric: two people can in principle be conceptually aligned in the absence of a shared (or even any) language. Sharing a language, however, necessitates at least *some* conceptual alignment.^2^ To see why, imagine asking participants to arrange colors according to their similarity. Participants who relied on a common set of color categories would produce more similar arrangements (blues all together; reds all together) than those who were not constrained by the categories. Not only should less categorical participants be less aligned with one another, there is reason to think that difficulties with name-based categorization leads to low alignment with oneself across time. For example, a patient with severe anomia but unimpaired perceptual processing tasked with arranging colors by similarity, produced sorts not only substantially different from those made by a control group, but also with himself, producing different arrangements each time he sorted (Roberson et al., 1999).^3^ Please see ‘labels_align_perturb_exp1.ipynb’ under Supplementary Materials for Python code for generating and perturbing the shapes.^4^ We switched to online data collection because of the COVID-19 pandemic.^5^ We thank an anonymous reviewer for pushing us on this point.^6^ Subsequent studies showed that much younger children are able to form categories on the basis of labels, e.g., Nazzi and Gopnik (2001) showed that 20-month olds (but not 16-month-olds) were able to use labels to form categories comprising arbitrary collections of items. Using a more implicit looking-time task, Plunkett et al., (Plunkett et al., 2008) showed that labels can be used to override visual similarity in 10-month old infants. Labeling a stimulus continuum with two labels led infants to split it into two, while using a single label led to infants unitizing the items into a single category. We thank an anonymous reviewer for encouraging us to expand on the links between the present work and developmental studies in more detail.

## Figures and Tables

**Figure 1S.**
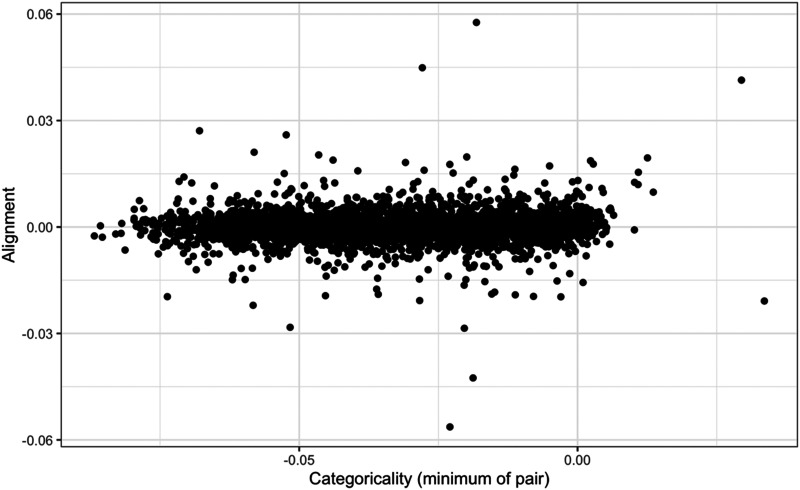
Relationship between pairwise categoricality (minimum of pair) and alignment for simulated participants (n = 3000) with completely random item placements.

**Figure 2S.**
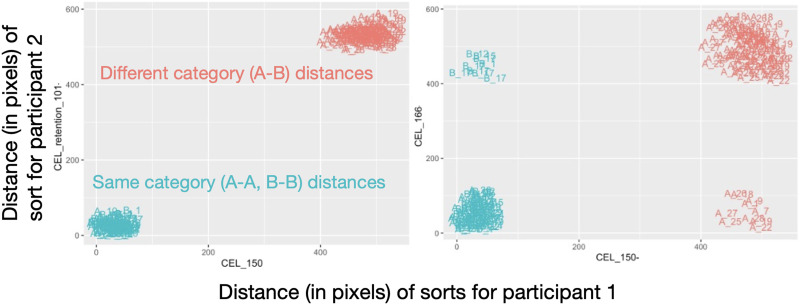
Pairwise distances between placed items for two pairs of subjects. Left shows a pair with extremely high alignment (r = .98). Right shows a pair with slightly lower alignment (r = .78). Color depicts distance type: different category (red), same-category (cyan).

**Figure 3S.**
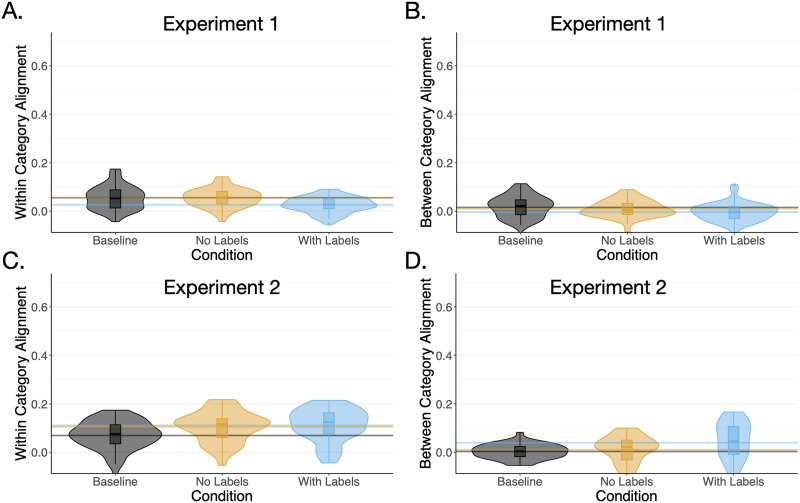
Condition differences for within-category alignment (A, C) and between category alignment (B, D). Scale is kept the same as Figure 6 (Exp. 1) and Figure 11 (Exp. 2) for ease of comparison.

## APPENDIX

### Multimember Analyses of Alignment

Please see 2_sort_task/supp_multimember_models.html (or.Rmd) for syntax and full output.

#### Experiment 1.

Using the more conservative approach to quantifying differences in alignment using *lmerMultiMember*, we find significantly greater alignment in the *With Labels* condition compared to *Baseline*, t = 2.41, and marginally higher alignment in *With Labels* compared to *No Labels*, t = 1.81.

#### Experiment 2.

We observed a similar, though slightly weaker pattern here: significantly greater alignment in the *With Labels* condition compared to *Baseline*, t = 2.17, but not significantly higher alignment in *With Labels* compared to *No Labels*, t = 1.23.

#### Experiments 1 and 2 Combined.

Combining Experiment 1 and 2 provides the extra power to observe significantly higher alignment in *With Labels* compared to *Baseline*, t = 3.18, and significantly higher alignment in *With Labels* compared to *No Labels*, t = 2.1.

Notice that high alignment is achieved almost entirely by having participants sort A’s with A’s and B’s with B’s such that within-category distance is smaller than between-category distance. Sorts that have this in common will have high alignment even if their arrangements of, e.g., A’s in their A cluster are quite different. Shuffling the cyan points does little to lower the correlation shown in Figure 2S.

### Alignment and Categoricality Are Not Related in Random Sorts

This analysis raises the question of whether alignment computed solely from within-category or solely from between-category distances show condition differences. Figure 3S visualizes these effects. To our surprise, within-category alignment was significantly lower in the *With-Label* condition of Experiment 1 than in the *No-Label* condition b = .029, t = 3.19, p = .002) and the Baseline condition (b = .03, t = 3.32, p = .002). Similarly, the *With-Labels* condition had slightly (but significantly) lower between-category alignment, though only compared to the *Baseline* condition (b = .02, t = 2.12, p = .04). These results show that a participant sorting the shapes after associating them with labels may resort to relying on category membership (“geks” go here, “talps” go there) without taking into account the finer-grained category structure. Their more aligned sorts at the category level appear to be accompanied by more idiosyncratic sorting at the local level.

### Within-Category vs. Between-Category Alignment

In contrast, Experiment 2, which used more familiar and easier-to-name stimuli showed a different pattern. Within-category alignment was significantly lower in the *Baseline* condition compared to both the *No-Labels* condition (b = .04, t = 2.46, p = .02) and the *With-Labels* condition (b = .04, t = 2.99, p = .004). Between-category alignment was significantly higher for the *With-Labels* condition compared to both the *Baseline* condition (b = .04, t = 2.65, p = .009) and the *No-Labels* condition (b = .03, t = 2.06, p = .04). These results suggest that using overtly presented labels to refer to previously familiar categories may lead to greater reliance on category-prototypes (e.g. sorts in which the more prototypical squares and circles end up ‘seeding’ the clusters formed during the sort).
